# Have Smooth-Faced Pluggers Any Advantage over Serrated?

**Published:** 1874-11

**Authors:** N. W. Williams

**Affiliations:** Geneva, Switzerland


### HAVE SMOOTH-FACED PLUGGERS ANY ADVANTAGE OVER SERRATED?

BY DR. N. W. WILLIAMS, GENEVA, SWITZERLAND.

*Read before the American Dental Society of Europe.*

It is quite evident to any one at all conversant with the progress of dentistry, that a vast improvement has been made in the mode of filling teeth within the last ten or fifteen years. The advent of adhesive gold and serrated instruments was the beginning of a great and important reform in the manner of filling the teeth. But we all remember how highly adhesive and harsh was the gold, how terribly deep and sharp were the serrations, and how miserable were some of the fillings. Both gold and fillings have undergone a gradual improvement, till now, we have nearly that which is desirable. When the

Varney instruments first made their appearance, they were adopted quite slowly by the profession, because the length of time the deeply serrated points had been in use, had engendered a belief in their thorough efficiency that was not easily shaken. Wherefore the utility of these new instruments, with the delicately serrated points, was regarded as somewhat doubtful, and they were tested with much hesitancy. But they have now taken a place as instrumeuts of high excellence, for the unanimous voice of the profession has pronounced them to be infinitely superior to those of earlier date. From these instruments, with the exquisitely fine serrations, it was but a step, though a bold one, to instruments with no serrations.

Reasoning from the admitted fact, that instruments increase in excellence as their serrations decrease in size, a few have deduced the conclusion that with a smooth-faced instrument the ultimate degree of perfection is approximated. In filling a tooth, the theory is to compress the numerous small particles of gold into a single mass of high density. Now, to accomplish this end, the instruments must be of such size, shape and finish as that they shall consolidate the gold, and at the same time spread or force it toward the walls or margin. If you will experiment with a piece of gold and a serrated instrument, you will find that when the gold is struck by the instrument, though it is consolidated, it is also contracted, drawn in toward the instrument rather than forced out toward a wall or margin; it is an effect of this natural tendency of serrated points to contract the gold, that many fillings are found to be encircled by a dark line. On the other hand, if you will perform the same experiment, using a smooth pointed instrument, you will find that while the gold is consolidated equally well, if not better, it is also spread or forced out towards a wall or margin, a result absolutely necessary to the permanent preservation of a tooth. It is in this marked difference in the result between the action of the smooth point and the serrated, the one consolidating and expanding in the sense of spreading out, the other consolidating but contracting, that there lies the advantage of the one over the other, which is claimed for smooth points by the few who have for several years used them with eminent success. Fillings that have been made with smooth points are to-day just as beautiful as they were at the time they were put in. No tiny pits, no discolor ation about the margin, no loosening of the gold at the edges is discoverable. They remain perfect, a delight to the artistic eye of the dentist. As to the shape of these instruments, at the beginning they were made flat on the surface and were highly polished, but it was found that they were very liable to slip, and that they left the gold so glaring that the eyes become painful after a few hours work. The points are now oval, somewhat like an instrument for counter sinking, perfectly smooth, but not polished. The tendency to slip is overcome and the bright glare is avoided.

